# Sustained metabolic dysregulation and the emergence of diabetes: associations between HbA1c and metabolic syndrome components in Tunisian diabetic and nondiabetic groups

**DOI:** 10.1186/s40101-024-00365-4

**Published:** 2024-07-20

**Authors:** Adriana Wisniewski, Alicia M. DeLouize, Tian Walker, Somnath Chatterji, Nirmala Naidoo, Paul Kowal, J. Josh Snodgrass

**Affiliations:** 1 Department of Anthropology, University of North Carolina at Chapel Hill, Chapel Hill, USA; 2 Global Health Biomarker Lab, Department of Anthropology, University of Oregon, Eugene, USA; 3 World Health Organization, Geneva, Switzerland; 4 Centre for Women’s Health Research, University of Newcastle, Callaghan, Australia; 5 Center for Global Health, University of Oregon, Eugene, USA; 6 Global Station for Indigenous Studies and Cultural Diversity, Hokkaido University, Sapporo, Japan

**Keywords:** Metabolic syndrome, Diabetes, Glycated hemoglobin, HbA1c, Noncommunicable disease, Global health, Preventative medicine

## Abstract

**Introduction:**

Metabolic Syndrome (MetS), diabetes, and other noncommunicable diseases (NCDs) have been a major focus of research in recent decades as the prevalence of these conditions continues to rapidly increase globally. However, the timing and patterns of development from metabolic risk factors to disease states are less well understood and are especially critical to understand in low- and middle-income countries (LMICs) and populations undergoing epidemiological transitions.

**Methods:**

Nationally representative sociodemographic, anthropometric, and point-of-care biomarker data from the 2016 Tunisian Health Examination Survey (*n = *8170) were used to determine the prevalence of diabetes and MetS components in Tunisia and to investigate associations between glycated hemoglobin (HbA1c) and MetS components (blood pressure [BP], HDL cholesterol [HDL], triglycerides [TG], and waist circumference [WC]) in participants aged 15-97 years old. To better understand how sustained metabolic dysregulation and disease states impact these associations, diabetic and nondiabetic groups were analyzed separately.

**Results:**

The overall prevalence of diabetes based on measured HbA1c was 18.2%. The diabetic groups had a higher prevalence of each individual MetS component, and significantly higher (BP, TG, WC, and HbA1c) and lower (HDL) values than the nondiabetic groups. Yet, there were a higher number of significant associations between HbA1c and MetS components found in nondiabetic women and men when compared to diabetic women and men. HbA1c was positively associated with the cumulative number of MetS components, irrespective of diabetes status in men and women.

**Conclusions:**

The prevalence of both diabetes and MetS components (particularly low HDL cholesterol and elevated TG) is high among the Tunisian population. More MetS components were associated with HbA1c in nondiabetic individuals, showing a strong connection between the development of MetS components and diabetes. However, once the diabetes disease state manifests, there is more variability in the relationships. These results show the potential for HbA1c to be an indicator of metabolic health below clinical disease cutoffs, which may allow insights into the physiological changes that precipitate the emergence of diabetes.

## Background

In recent decades, the prevalence of noncommunicable diseases (NCDs) has increased worldwide as many countries undergo rapid social, demographic, and epidemiological transitions. Currently, NCDs cause 74% of deaths globally, with 77% of deaths due to NCDs occurring in low- and middle-income countries (LMICs) [1]. Metabolic dysregulation results from physiological changes that disrupt the body’s metabolism. One way to assess metabolic dysregulation is Metabolic Syndrome (MetS). MetS is not characterized by a single metabolic abnormality; rather, it encompasses a group of metabolic abnormalities that contribute to increased risk for NCDs, such as cardiovascular disease (CVD) and type 2 diabetes (T2D) [2]. There is no single pathway or process between shared metabolic risk factors and NCD development. Instead, metabolic dysregulation can lead to a variety of NCDs and is influenced by variations in the physiological patterns and timelines of processes from metabolic dysregulation to energy imbalances that eventually culminate in the development of disease states. Steps can be taken to alter lifestyle to prevent NCD development or severity. Yet once a disease state is achieved, metabolic dysregulation continues to increase risk for other NCDs and comorbidities.

The purpose of the present study is to develop a better understanding of how a disease state, like diabetes, impacts the prevalence and types of MetS components and overall metabolic health within a population and to assess the ability of hemoglobin A1c (HbA1c), also known as glycated hemoglobin, to indicate metabolic health beyond glucose metabolism, including below clinical cutoffs used to diagnose disease. HbA1c has been used as a supplement to or in place of fasting blood glucose as a hyperglycemia criterion for MetS detection [3, 4]. Rather than analyzing HbA1c as a potential component of MetS, we examine HbA1c as an outcome variable by evaluating the relationships between HbA1c and metabolic syndrome components in people with and without diabetes. There are known and well-studied relationships between HbA1c and MetS and its components of blood pressure, HDL cholesterol, triglycerides, and waist circumference, as NCDs and associated risk factors have been a large focus over the past few decades. Previous studies have found positive associations between MetS components and HbA1c in nondiabetic populations [5, 6]. Associations in diabetic populations have also been examined [7, 8]. Studies have also analyzed risk for metabolic syndrome and other related conditions in diabetics vs nondiabetics, associations with HbA1c based upon the presence or absence of another disease (e.g. cardiovascular disease), or clustering of components in diabetic vs nondiabetic populations, but the impact of MetS on HbA1c has not been well examined when separating a population based on the presence of diabetes [9–11]. Thus, we examine the degree to which MetS components may impact HbA1c differently based upon disease state status (i.e., diabetic vs. nondiabetic) within one population (Tunisia) [12].

### Defining and exploring metabolic syndrome and diabetes

There is no consensus on the defining criteria for MetS, with multiple approaches available; however, in general MetS is characterized by a combination of glucose intolerance/insulin resistance, central obesity, hypertension, and dyslipidemia [13]. In this way, it is closely related to diabetes, CVD, and other NCDs, yet the variation between individuals in the relationships among components has not often been differentially evaluated in people with and without an NCD. The timing of the development of MetS components and numerous elements of MetS pathophysiology, including glucose and fat metabolism, insulin, adipocyte-derived hormones, and cytokines, complicate the sequential and clustering relationships of components, and the physiological pathways that connect individual factors are all aspects of MetS that require more research.

Diabetes has been shown to be particularly closely associated with MetS. While the causes of MetS are not fully understood, there are strong associations between the development of MetS and the presence of insulin resistance, suggesting a commonly shared pathway that links MetS to an increased risk for T2D [14]. Some MetS diagnostic criteria require an element of insulin resistance for MetS diagnosis [1]. The global prevalence and burden of diabetes have been rising rapidly in both LMICs, and high-income countries (HICs), and are projected to grow by 20% in the next decade [15]. Both type 1 diabetes (T1D) and T2D are characterized by hyperglycemia, high levels of glucose within the blood, and alterations to beta cell function, insulin secretion, cellular response to insulin, or both [16, 17]. The impact of this is inefficient uptake and metabolism of glucose by the body’s cells, which results in increased levels of glucose in the blood and increased cellular fat and protein metabolism to compensate for reduced glucose metabolism [16]. Once the disease state is achieved, those with diabetes are at higher risk for other metabolic risk factors (MetS components) due to changes in how the body uses and stores different types of energy (lipids and proteins) [18, 19].

Although diabetes has been a large focus of scientific and medical research for decades, much is still unknown about the specific physiological pathways leading to disease development and progression. Diabetes is a disease with multifactorial etiology, meaning there is not just one pathway leading to diabetes development, and the exact pathways and relationships between pathways are not well understood. There is additional complexity due to the considerable variation in disease progression between individuals. For example, sustained high blood sugar levels, commonly present in individuals with undiagnosed diabetes or poorly managed diabetes, can increase the risk for CVD [20, 21]. Diabetes can also increase risk for other NCDs, including obesity, hyperlipidemia, chronic kidney disease, stroke, cognitive decline, decreased quality of life, and premature death [22, 23]. The links between diabetes and other NCDs are in part due to connections to obesity, chronic inflammation, oxidative stress, and insulin resistance [24, 25]. Obesity, more specifically, central adiposity (measured using waist circumference [WC]) and associated chronic systemic inflammation, are also believed to be closely related to both MetS factors, diabetes, and other NCDs.

### Defining and using HbA1c

Over the past several decades there has been an increase in the use of HbA1c for diabetes diagnosis and management. HbA1c measures the percentage of a hemoglobin compound produced by an irreversible reaction between a glucose molecule and a hemoglobin molecule within the blood [26]. HbA1c formation is proportional to the concentration of glucose in the blood. Red blood cells, which bind to hemoglobin, have an average lifespan of 120 days. Thus, HbA1c levels reflect the average blood glucose level over the previous 2-3 months [26]. HbA1c is increasingly being utilized in the screening for and diagnosis of diabetes, to evaluate disease progression, and to monitor treatment and management of the disease [27, 28]. A benefit of using HbA1c is the insight into metabolic health over a longer time frame compared to fasting glucose values and other single time point biomarkers; furthermore, HbA1c can be measured in individuals who are not fasted.

### Beyond metabolic risk factors

MetS and diabetes are also influenced by environmental, genetic, and lifestyle risk factors. Environmental factors, including early life conditions, viral infection, immune system and gut microbiome development, and diet, have all been found to influence risk for disease [29]. Recently, studies have shown connections between SARS-CoV-2 infection and increased prevalence of T1D and T2D, increased rates of diabetic ketoacidosis at the time of T1D diagnosis, and worse COVID-19 outcomes in those with diabetes [30–32]. Sedentary habits and changes to diet, such as increased carbohydrate consumption, as seen during pandemic lockdowns, have been associated with an increased risk of T2D development [33]. Genetic and epigenetic influences on the risk of developing diabetes and multiple aspects of MetS have also been well-studied [34, 35]. Multiple genome-wide association studies have led to the identification of multiple genes involved in risk for T2D [35]. Gene-environment interactions such as with increased adiposity, may alter T2D risk directly by influencing insulin action or secretion, but also altering environmental interactions [35]. Epigenetic changes due to the maternal environment and conditions during early infancy can alter risk for chronic disease (including diabetes and metabolic syndrome) throughout the life course [35]. Beyond individual-level physiology, more research is also needed to understand better population-level factors that may contribute to MetS and its relationships to NCDs.

The present study provides epidemiological data on the prevalence of clinically recognized (diagnosed) and undiagnosed diabetes and MetS components (blood pressure [BP], HDL cholesterol levels [HDL], blood triglyceride [TG] levels, and waist circumference [WC]) in Tunisia using a nationally representative sample from the Tunisian Health Examination Survey (THES). Additionally, it investigates relationships between MetS components and HbA1c levels in diabetic and nondiabetic Tunisian adults to evaluate how many and which specific components of MetS affect HbA1c levels within two different timelines in terms of metabolic dysfunction. The relatively novel approach of separating groups by disease state thereby also provides insight into the potential for HbA1c to indicate the impact of achieving a disease state (i.e., diabetes), while also assessing metabolic changes and risk in those who do not meet or exceed clinical cutoff values for diabetes diagnosis. This allows the measurement of subtle changes to glucose metabolism in nondiabetic individuals, a concept not common within the broader diabetes literature.

Tunisia is a lower-middle income country located in Northern Africa. It has been undergoing demographic, epidemiologic, and nutritional transitions in recent decades. As a result of these transitions, NCDs are an increasing health concern. According to the WHO in 2016, NCDs were responsible for 86% of all deaths in Tunisia (44% from cardiovascular diseases, 12% from cancer, 4% from chronic respiratory diseases and 5% from diabetes) as well as 16% of premature deaths between 30-70 years [36]. With a large burden of NCDs and a dynamic social and economic landscape, assessing metabolic health and risk in Tunisia may contribute valuable insight into groups within the country that may be at higher risk for developing NCDs. Additionally, this study contributes to a growing body of literature with increased attention to health within lower- and middle-income countries on a global scale, as well as literature focused on Tunisia specifically.

### Hypotheses

We hypothesize that there will be positive associations between HbA1c and 1) each individual component of MetS and 2) the cumulative number of MetS components among both diabetic and nondiabetic groups.

## Methods

### Tunisian health examination survey

The Tunisian Health Examination Survey (THES) is a nationally representative study used to monitor population health and provide supporting evidence for health policy and strategy development. The primary purpose was to analyze the state of population health and its determinants, disease consequences, and the use of point-of-care testing (POCT). Some non-private dwellings, such as prisons, hospitals, hospices, dementia care units, and some remote areas, were excluded from the survey population. The THES used a stratified multistage cluster sampling design to monitor population health and provide supporting evidence for health policy and strategy development. Households are selected at random, and one individual per household is then selected through a procedure using the Kish table method.

The present study uses sociodemographic characteristics, anthropometrics, biomarkers, risk factors, preventative health behaviors, and chronic conditions data from the individual THES questionnaire. Trained interviewers performed face-to-face computer-assisted personal interviews and collected anthropometric, biomarker, and blood pressure measurements. The interviews took place from January to May 2016 in the participants’ homes.

### Participants

Households (*n* = 5079) were selected in 3 stages with 7 regions, 24 governorates, and 351 districts included. This study analyzes data from (*n* = 8,170) THES participants after accounting for exclusions due to incomplete data (*n* = 964), pregnant individuals (*n* = 138), and individuals with out-of-range biomarker data (*n* = 904) (i.e., defined as above or below the following measurement ranges: Total cholesterol [100-400 mg/dL], HDL cholesterol [20-120 mg/dL], TG [50-500 mg/dL], blood glucose [20-600 mg/d], HbA1c [4.0-13.0%]).

The sample was 52.3% women. Participants ranged from 15 to 97 years old (*M* = 48.01, *SD* =17.33), and the age range for individuals (adults) to participate in the study was determined by Tunisian-based researchers. Formal education ranged from 0 to 34 years (*M *= 7.44, *SD* = 5.97). Mean education was particularly low because 25.1% of the sample had no formal education. Most participants were married (73.7%) as opposed to being never married, widowed, or divorced, and the majority of people lived in urban settings (64.5%). The monthly household income ranged from 0 – 12,000 Tunisian dinars (*M* = 699.56, *SD* = 766.48).

### Sociodemographic measures

Gender, age, marital status, education, urban/rural location, and perceived health were determined using standard survey measures. Questionnaire responses regarding diabetes, dyslipidemia, and hypertension, answers to symptom and medication use questions and participant demographic information were used in the present study.

### Anthropometrics and blood pressure

#### Waist and hip circumference

Waist and hip circumference were measured using a flexible measuring tape (Gulick II Measuring tape model – 67020). WC was measured at the top of the iliac crest. Hip circumference was measured around the maximum circumference of the participant’s buttocks. Both waist and hip circumference measurements were recorded to the nearest 0.1 cm.

#### Blood pressure

Blood pressure was measured using an automatic wrist blood pressure monitor (Omron M6 Comfort; Omron Healthcare, Kyoto, Japan). Three blood pressure measurements were taken on the participants’ left wrist, which was held steady at the approximate level of the heart. All systolic and diastolic measurements were recorded in units of mmHg. Accuracy of the device is ±3 mmHg for pressure and ±5% of display reading for pulse.

#### Biomarker collection

All biomarker data were collected using finger prick capillary blood samples and POCT devices. Total cholesterol (100-400 mg/dL), HDL cholesterol (20-120 mg/dL), TG (50-500 mg/dL) and blood glucose (20-600 mg/dL) were measured using the CardioChek PA system (Polymer Technology Systems, Inc., Whitestown, IN) and values were recorded in mg/dL. The CardioChek PA system meets US National Cholesterol Education Program (NCEP) guidelines for accuracy and precision.

Percent hemoglobin A1c (HbA1c) (4.0-13.0%) was measured using the A1CNow+ device (Polymer Technology Systems, Inc., Whitestown, IN). The overall imprecision of the A1cNow+ system is 3.00% CV at the low level and 4.02% at the high level, meeting National Glycohemoglobin Standard Program certification. The accuracy of the A1c+Now system on average was 99% from fingerstick (capillary) comparative testing, the accuracy was on average 99.7% from venous comparative testing.

#### Diabetes

Individuals with self-reported diabetes diagnosis or those with measured HbA1c values ≥ 6.5% were included in the diabetic group. An HbA1c of 6.5 was used as part of the criteria for the diabetic group as 6.5 is the widely accepted clinical cutoff value for diabetes diagnosis [37]. Those with measured HbA1c values < 6.5% and no self-reported diabetes diagnosis were placed in the nondiabetic group.

#### Metabolic syndrome

There is not one universally accepted MetS measurement definition for use in clinical settings; instead, recognition of MetS is generally based on well-recognized signs, including abdominal obesity, elevated TG, low HDL cholesterol, elevated BP, and/or high plasma glucose [13]. The MetS components analyzed for the present study included BP, HDL cholesterol, TG, and WC. Blood glucose was excluded from our analyses due to not all participants having fasted when data were collected and that both measures (blood glucose and HbA1c) ultimately measure glucose, though at different time scales.

We used the National Cholesterol Education Program (NCEP) Adult Treatment Panel (ATP) III diagnostic criteria for MetS (Table 1), which is one of the most widely used. It does not require one specific component of MetS for diagnosis and therefore does not incorporate any preconceived notion of an underlying cause of MetS (such as insulin resistance or obesity) [38].

**Table 1:** ATP III MetS criteria

**NCEP ATP III (2005)**		
**Required**		None
**Hyperglycemia**	Blood glucose	> 100.8 mg/dl (5.6 mmol/L)^a^
**Dyslipidemia**	HDL Cholesterol	< 40 mg/dl (1.0 mmol/L) (men)^a^ < 50 mg/dl (1.3 mmol/L) (women)^a^
	Triglycerides	> 150 mg/dl (1.7 mmol/L)^a^
**Obesity**	Waist Circumference	> 102 cm (men) > 88 cm (women)
**Hypertension**	Blood Pressure	> 130/85 mmHg^a^

^a^ Or drug treatment for specific criteria

### Statistical analyses

R version 4.3.0 was used for all statistical analyses and the alpha level was set to 0.05. All statistical assumptions were checked before analysis. HbA1c values were log-transformed for linear analysis due to non-normality (skew = 2.61, kurtosis = 8.29). Monthly household income was log-transformed due to non-normality (skew = 4.73, kurtosis = 39.02). Following standard practice, men and women were analyzed separately because of divergences in metabolic processes and risk factors closely related to sex, which are also reflected in differences in MetS criteria. However, it should be noted that sex differences in biological measures are typically minor, and distributions generally overlap considerably, with environmental factors having major effects [39]. In our analyses, men and women were also further divided into diabetic and nondiabetic groups.

All statistics were run separately on the four groups: nondiabetic and diabetic men and women. Descriptive statistics on HbA1c and MetS components are presented along with inferential statistics on their pairwise comparisons. Spearman’s rank correlations were performed to test associations between untransformed HbA1c and the total number of MetS components. Multiple linear regressions with maximum likelihood estimation were used to evaluate the partial correlations between each MetS component and log HbA1c, and then also the cumulative number of MetS components and log HbA1c. Fully adjusted models excluded participants who were on medications for diabetes and controlled for age, years of education, urban/rural household location, and monthly household income (Tunisian dinars). The exponentials of coefficients are presented.

## Results

Diabetes poses a significant burden to the health of people living in Tunisia with an estimated 17.6% of women and 18.8% of men from this study with the condition (Table 2). Combined, 18.2% of participants had diabetes. In the nondiabetic population, the mean HbA1c value was 5.5% in both women and men. In the diabetic population, the mean HbA1c value was 7.9% in women and 8.1% in men (Table 2).

**Table 2:** Prevalence of diabetes in diabetic and nondiabetic women and men, and average HbA1c values

	**Diabetic**		**Nondiabetic**			
	***n***	***M ± SE***	***n***	***M ± SE***	***t***	
**Women**	752 (17.6%)	7.94 ± 0.06	3517 (82.4%)	5.46 ± 0.01	-44.87	***
**Men**	734 (18.9%)	8.11 ± 0.07	3167 (81.1%)	5.45 ± 0.01	-44.38	***

*** *p* < 0.001

Within the diabetes groups, 60.0% of women and 57.9% of men had reported previous clinical diabetes diagnoses; the rest had HbA1c values greater than or equal to 6.5% without a diagnosis. Of those with a diabetes diagnosis, 91.4% of women and 92.2% of men were on medication (insulin, tablets [e.g., Metformin or Amaryl], or both insulin and tablets) (Table 3). Those without a diagnosis did not utilize medication for diabetes.

**Table 3:** Prevalence of diabetes medication use and average HbA1c in diabetic men and women

	**Women**		**Men**	
	***n***	***M* ± SE**	***n***	***M* ± SE**
**No Medication**	39 (8.6%)	7.25 ± 0.31	33 (7.8%)	7.41 ± 0.39
**Insulin**	78 (17.3%)	8.83 ± 0.20	87 (20.5%)	8.81 ± 0.21
**Tablet**	296 (65.6%)	8.08 ± 0.11	269 (63.3%)	8.16 ± 0.12
**Insulin & Tablet**	38 (8.4%)	9.55 ± 0.25	36 (8.5%)	8.60 ± 0.30
**NA (No diagnosis)**	301 (-)	7.46 ± 0.08	309 (-)	7.90 ± 0.10

Diabetic women with a diagnosis, on any type of medication for diabetes, had significantly higher mean HbA1c values than those not taking medication (*M* = 7.25). Those on both insulin and tablets had the highest mean HbA1c values (*M* = 9.55, *t* = -6.31, *p* < 0.001) followed by insulin (*M* = 8.83, *t* = -4.54, *p* < 0.001), and then by tablets (*M* = 8.08, *t* = -2.77, *p* < 0.01) (Table 3).

Diabetic men with a diagnosis on insulin or insulin and tablets had significantly higher mean HbA1c values than those not taking medication (*M* = 7.41). Those on insulin had the highest mean HbA1c values (*M* = 8.81, *t* = -3.32, *p* < 0.01), followed by those on insulin and tablets (*M* = 8.60, *t* = -2.80, *p* < 0.01), and then those on tablets (*M* = 8.16, *t* = -2.02, *p* > 0.05) (Table 3).

The prevalence of individual MetS components BP, HDL, TG, and WC were calculated for diabetic and nondiabetic women and men, as well as the total prevalence by gender (Table 4).

**Table 4:** Prevalence of metabolic syndrome components in diabetic and nondiabetic women and men

		**Women**			**Men**		
		**Diabetic**	**Nondiabetic**	**Total**	**Diabetic**	**Nondiabetic**	**Total**
**MetS Component**	**BP**	423 (56.6%)	932 (26.5%)	1355 (31.8%)	361 (49.3%)	749 (23.7%)	1110 (28.5%)
	**HDL**	462 (61.4%)	1739 (49.4%)	2201 (51.6%)	455 (62.0%)	1592 (50.3%)	2047 (52.5%)
	**TG**	572 (76.1%)	1884 (53.6%)	2456 (57.5%)	556 (75.7%)	1974 (62.3%)	2530 (64.9%)
	**WC**	580 (82.3%)	1949 (56.6%)	2529 (61.0%)	273 (38.4%)	535 (17.2%)	808 (21.2%)

Components determined by values that exceed ATP III Metabolic Syndrome criteria thresholds (Table 1)

The prevalence of the cumulative number of MetS components and associated average HbA1c values by number of components for diabetic and nondiabetic men and women are shown in Table 5.

**Table 5:** Prevalence of and average HbA1c values by cumulative number of metabolic syndrome components

**# of MetS Components**		**Women**		**Men**	
		**Diabetic**	**Nondiabetic**	**Diabetic**	**Nondiabetic**
**0**	***n***	25 (3.6%)	462 (13.4%)	55 (7.8%)	547 (17.6%)
	**HbA1c *M* ± *SE***	7.62 ± 0.32	5.34 ± 0.02	7.87 ± 0.26	5.30 ± 0.02
**1**	***n***	63 (9.0%)	915 (26.6%)	116 (16.4%)	997 (32.1%)
	**HbA1c *M* ± *SE***	7.54 ± 0.19	5.41 ± 0.02	7.84 ± 0.16	5.41 ± 0.01
**2**	***n***	173 (24.6%)	997 (29.0%)	232 (32.7%)	1031 (33.2%)
	**HbA1c *M* ± *SE***	8.01 ± 0.14	5.44 ± 0.02	8.01 ± 0.13	5.49 ± 0.01
**3**	***n***	238 (33.9%)	770 (22.4%)	209 (29.5%)	420 (13.5%)
	**HbA1c *M* ± *SE***	7.83 ± 0.11	5.57 ± 0.02	8.33 ± 0.14	5.55 ± 0.02
**4**	***n***	204 (29.0%)	295 (8.6%)	112 (3.6%)	112 (3.6%)
	**HbA1c *M* ± *SE***	8.14 ± 0.13	5.63 ± 0.03	8.37 ± 0.18	5.59 ± 0.04
***ρ***		0.09	0.19	0.12	0.18
***p***		0.02 *	< 2.2e-16 ***	0.002 **	< 2.2e-16 ***

Components determined by values that exceed ATP III Metabolic Syndrome criteria thresholds (Table 1)

^*^ *p* < 0.05; ** *p* < 0.01; *** *p* < 0.001

Associations between untransformed HbA1c and the cumulative number of MetS components were assessed using Spearman’s rank correlation. 

**Fig. 1:**
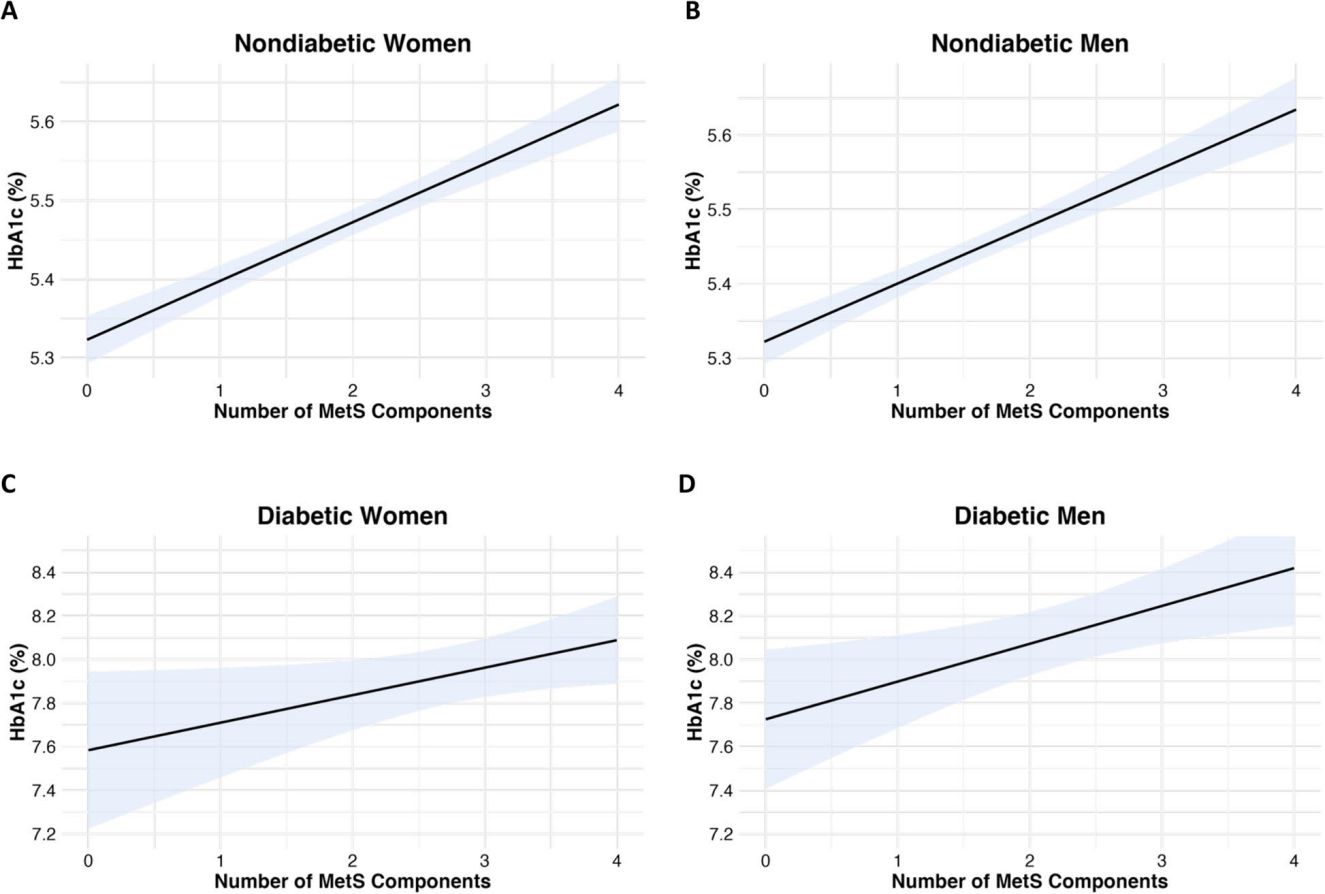
**A-D** Correlation graphs for the Number of MetS components and HbA1c (%).Note. Nondiabetic women (A, *p* < 0.001), nondiabetic men (B, *p* < 0.001), diabetic women (C, *p* < 0.05) and diabetic men (D,* p* < 0.05). Significance was determined using Spearman's rank correlations

HbA1c was positively correlated with the total number of MetS components in all groups: nondiabetic men (*ρ =* 0.18, *p* < 0.001) and women (*ρ* = 0.19, *p* < 0.001), and diabetic men (*ρ* = 0.12, *p* < 0.05) and women (*ρ* = 0.09, *p* < 0.05) (Table 5; Fig. 1A-D).

In women, the average values for diastolic BP, systolic BP, TG, and WC were significantly higher, and the average value of HDL cholesterol was significantly lower, in the diabetic population compared to the nondiabetic population (Table 6).

**Table 6:** Average values of  metabolic syndrome components in nondiabetic and diabetic women

	**Diabetic** ***M* ± *SE***	**Nondiabetic** ***M* ± *SE***	***t***	
Average Systolic Blood Pressure	139.94 ± 0.79	125.46 ± 0.34	-16.80	***
Average Diastolic Blood Pressure	84.58 ± 0.41	81.94 ± 0.19	-6.93	***
HDL	50.50 ± 0.52	52.07 ± 0.25	2.72	**
Triglycerides	217.78 ± 3.42	176.18 ± 1.43	-11.23	***
Waist Circumference	100.00 ± 0.53	91.03 ± 0.26	-15.27	***

**p < 0.01; ***p < 0.001

In men, the average values of diastolic BP, systolic BP, TG, and WC were significantly higher, and the average value of HDL cholesterol was significantly lower in the diabetic population compared to the nondiabetic population (Table 7).

**Table 7:** Average values of Metabolic Syndrome Components in diabetic and nondiabetic men

	**Diabetic and/or HbA1c ≥ 6.5** ***M* ± *SE***	**Nondiabetic** ***M* ± *SE***	***t***	
Average Systolic Blood Pressure	140.97 ± 0.78	129.38 ± 0.32	-13.71	***
Average Diastolic Blood Pressure	83.29 ± 0.44	79.10 ± 0.19	-8.65	***
HDL	39.38 ± 0.46	41.89 ± 0.23	4.87	***
Triglycerides	226.83 ± 3.75	196.88 ± 1.71	-7.27	***
Waist Circumference	98.67 ± 0.52	90.25 ± 0.24	-14.68	***

*** *p* < 0.001

**Fig. 2:**
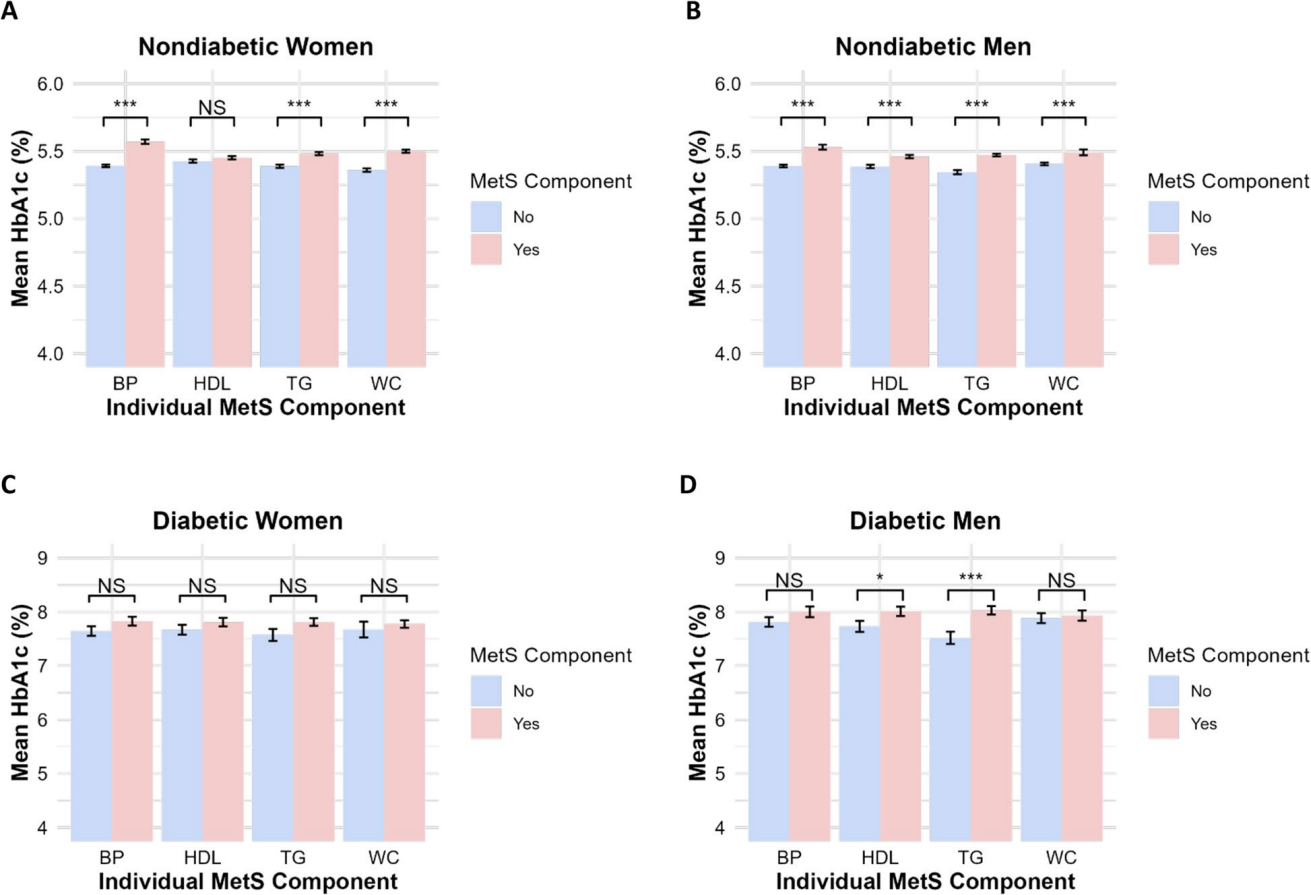
**A-D** Average HbA1c by the presence of Individual MetS components (BP, HDL, TG, and WC). Note. Nondiabetic women (**A**), nondiabetic men (**B**), diabetic women (**C**) and diabetic men (**D**). Significance was determined using *t-*tests with the log of HbA1c as the outcome. (“NS” *p* > 0.05; * *p* < 0.05; ** *p* < 0.01; *** *p* < 0.001)

Pairwise comparisons between the average HbA1c values for individuals with and without each MetS component were assessed in all four groups (non-diabetic and diabetic, men and women). In nondiabetic women log HbA1c was significantly associated with MetS components of BP (*t =* 9.45, *p* < 0.001), TG (*t =* 5.48, *p* < 0.001), and WC (*t =* 8.20, *p* < 0.001) (Fig. 2A). In diabetic women, no significant differences in HbA1c were observed with any MetS components (Fig. 2C). Significantly higher HbA1c values were observed in nondiabetic men with MetS components of BP (*t =* 6.85, *p* < 0.001), HDL (*t =* 4.22, *p* < 0.01), TG (*t =* 6.95,* p* < 0.001), and WC (*t* = 3.60, *p* < 0.001) (Fig. 2B). Significantly higher HbA1c values were observed in diabetic men with the MetS components of HDL (*t =* 2.09, *p* < 0.05) and TG (*t =* 3.65, *p* < 0.001) (Fig. 2D).

Multiple linear regressions were performed on four groups (women and men who were nondiabetic and women and men who were diabetic and did not take medications) to assess the association of metabolic variables with while also accounting for covariates of age, years of education, urban/rural location, and (monthly) household income (Table 8). Then linear regressions with the cumulative number of MetS components with the same covariates were also performed (Table 9).

**Table 8:** *β* Values for multiple regression predicting log HbA1c based upon individual MetS components and covariates (age, education, urban/rural location, and log monthly household income)

	**Women**		**Men**	
	Diabetic	Nondiabetic	Diabetic	Nondiabetic
BP	0.0075	0.011**	0.085***	0.0093*
HDL	0.010	0.0061	0.0012	0.014***
TG	0.023	0.0059	0.060*	0.017***
WC	0.020	0.011**	-0.027	0.0037
Age	-0.00016	0.012***	-0.00019	0.0011***
Education	-0.00033	-0.000023	0.021	0.00041
Rural	-0.028	-0.0027	-0.0014	0.0028
Household Income	0.0028	0.0025	0.0096	0.0047*
Adj. R Squared	-0.01	0.07	0.04	0.07
Log-likelihood	108.75	3183.02	55.53	2918.86

^*^ *p* < .05, ** p < .01, *** *p* < .001; The diabetic groups were restricted to individuals that were not taking medications

**Table 9:** *β* Values for Multiple Regression predicting log HbA1c based upon cumulative number of MetS components and covariates (age, education, urban/rural location, and log monthly household income)

	**Women**		**Men**	
	Diabetic	Nondiabetic	Diabetic	Nondiabetic
Number of MetS Components	0.015	0.0082***	0.033**	0.012***
Age	-0.00020	0.0012***	0.00021	0.0011***
Education	-0.00036	-0.00023	0.00077	0.00039
Rural	-0.028	-0.0031	0.023	0.0029
Household Income	-0.0025	0.0026	-0.0030	0.0045*
Adj. R Squared	-0.002	0.07	0.02	0.06
Log-likelihood	108.58	3182.07	49.70	2915.94

^*^ *p* < .05, ** p < .01, *** *p* < .001; The diabetic groups were restricted to individuals that were not taking medications

## Discussion

Our results provide unique insights into the prevalence of diabetes and MetS components in a large, nationally representative sample of Tunisian adults. Few studies on the prevalence of these conditions have been conducted in LMICs such as Tunisia. Population-level studies that are based on measured health data (rather than self-report) are extremely valuable as they expand beyond projections and estimates and give us objectively measured values without relying on participant memory or recall, or their access to healthcare. Relying solely upon clinical diagnoses for estimating diabetes prevalence would result in the exclusion of a large portion of the diabetic population due to lack of clinical diagnosis. We show that HbA1c has different relationships to MetS components based on diabetes disease status, which could stem from the metabolic regulation of varying components in the disease state.

### Diabetes prevalence and access to healthcare in Tunisia

As of 2021, the prevalence of diabetes in Tunisia in the literature was estimated to be 10.8% according to the International Diabetes Federation based upon a combination of oral glucose tolerance test (OGTT), fasting blood glucose (FBG), HbA1c, self-reported diabetes, and medical record/clinical diagnosis data from existing diabetes sources [15]. Our results show that 18.2% of our nationally representative sample from 2016 has diabetes, a value much higher than both the 2021 estimation for Tunisian adults and the 10.5% estimation of the global prevalence of diabetes [15]. Of those in our sample in the diabetic group, 40.0% of women and 42.1% of men who have HbA1c values greater than or equal to 6.5% do not have a formal diagnosis. This large proportion of the diabetic population being undiagnosed likely indicates a recent shift in the burden of disease, as well as a lack of screening resources for diabetes and access to healthcare. The substantially higher prevalence of diabetes documented in the present study has important social and economic implications for Tunisia.

Those without a diabetes diagnosis did not utilize medication. Of those with a diabetes diagnosis, 91.4% of women and 92.2% of men were on medication (insulin, tablets [e.g., Metformin or Amaryl], or both insulin and tablets), indicating generally good access to healthcare and medication once a diagnosis is received. This further supports the conclusion that the rapid increase in chronic disease prevalence is putting an increased burden on healthcare systems that are not fully prepared to address the growing prevalence of diabetes and, beyond that, NCD prevention and treatment in general.

Previous studies conducted using the THES data have indicated income-related inequalities in cardiovascular risk factors in those with a clinical diagnosis of diabetes [40]. Another study found healthcare access or refusal of health care in the Tunisian diabetic population was driven primarily by financial reasons (i.e., lack of health insurance) [41]. Furthermore, associations have been found between inequalities in healthcare and social determinants of health such as geographical location, gender, education, and SES [42]. We found that many participants did not have a diagnosis of diabetes, which is in line with previous research showing high rates of undiagnosed hypertension in the THES participants, in part due to a lack of disease education and barriers to healthcare access [43].

### Metabolic syndrome component prevalence in Tunisia

The prevalence of MetS components and population-specific patterning has not been thoroughly assessed in Tunisia at a population level. Results show that over half of Tunisian women met or surpassed the MetS cutoff values for HDL cholesterol, TG, and WC, and over half of Tunisian men met or exceeded the cutoffs for HDL cholesterol and TG. These results indicate increased susceptibility to metabolic risk factors related to lipid metabolism in higher proportions of the Tunisian population. Additionally, the results support that Tunisian women were more susceptible to central adiposity and obesity than men, as nearly 60% of Tunisian women have waist circumference values that surpassed the MetS cutoff, while only approximately 20% of men met or exceeded the cutoff. However, anthropometric dimensions are known to have population differences in cutoff points, and it is possible these values do not indicate health status. These findings provide insights into the components of MetS that may disproportionately affect the general Tunisian population.

Unsurprisingly, when assessing diabetic and nondiabetic groups separately, for both women and men, the diabetic groups had higher percentages of individuals with MetS components than the nondiabetic groups. Not only were MetS components more prevalent in the diabetic groups, but it is also essential to acknowledge that in both women and men, the diabetic groups had poorer metabolic health as indicated by statistically significant higher values for HbA1c, systolic and diastolic BP, TG, and WC, and lower HDL cholesterol values, supporting the view that diabetes impacts not only metabolic health related to glucose metabolism but also obesity and lipid metabolism, which may increase the risk for other NCDs like CVD.

The availability of information about which specific MetS components disproportionately affect the Tunisian population has the potential to provide insights that can benefit the general population and healthcare professionals as the country continues to undergo social, demographic, and epidemiological changes, and the NCD burden continues to grow.

### Hypothesis testing (diabetic vs. nondiabetic)

When testing our first hypothesis, if HbA1c was positively associated with individual MetS components using multiple regressions in diabetic (not on medication) and nondiabetic groups, it was surprising to find that in nondiabetic women the presence of MetS components of BP and WC were associated with significantly higher HbA1c, age was also positively associated with HbA1c in this model. The same was true for nondiabetic men with the components of BP, HDL cholesterol, and TG, in this model age and monthly household income were also significantly positively associated with HbA1c. This indicates that prior to reaching a diabetes disease state, HbA1c may be tied to metabolic components; these relationships remain relatively unexplored.

Meanwhile, this relationship between MetS component and HbA1c was not as strong in the diabetic groups. There were no significant associations in the diabetic women group between HbA1c and any MetS components or covariates. In the men, BP and TG were the only variables significantly associated with HbA1c, and they were positively associated. These results suggest that once the diabetic disease state was reached, the individual MetS components alone were not significantly associated with HbA1c, meaning having one component did not necessarily indicate increased HbA1c once it had surpassed the threshold of 6.5%. Alternatively, in the nondiabetic group, most MetS components were significantly positively associated with HbA1c, meaning below the 6.5% HbA1c threshold widely utilized for clinical diabetes diagnosis; HbA1c may provide additional insight into other forms of metabolic dysregulation apart from glucose metabolism or insulin resistance below a certain threshold or level of dysregulation.

When testing our second hypothesis, if HbA1c was positively associated with the cumulative number of MetS components, our multiple linear regression model results showed that the number of MetS components was significantly positively associated with HbA1c in diabetic men (not on medications) and both nondiabetic groups (men and women). This indicates that overall metabolic health worsens as indicated by the accumulation of more MetS components, there is an association with increased HbA1c. Thus, increased HbA1c values may be indicative of changes to not only glucose metabolism and/or insulin resistance that are known to be closely related to changes in insulin signaling, production, absorption, or a combination of those factors, but also the physiological systems closely related to the other MetS components.

While directional and causal relationships cannot be established due to a lack of longitudinal data, through this cross-sectional analysis provides insight into an important finding that suggest that managing and treating individual components may help reduce the cumulative number of components that may have further implications on metabolic health beyond the specific component being treated. It is important to note that while we found significance in the nondiabetic groups, and less in the diabetic groups, it does not mean that these physiological transitions and connections are not occurring in the diabetic population. Rather, the severity of the degree of dysregulation to glucose metabolism in the diabetic groups may be hindering our ability to assess other forms of metabolic dysregulation, resulting in a lack of significance in our analyses.

It is not surprising that age was positively associated with increases in HbA1c in some groups when assessing both the impact of individual and cumulative number of components, although the significant results were only found in nondiabetic women and men. This finding is consistent with a variety of studies of age and HbA1c in nondiabetic populations [44–47]. This association between age and increasing HbA1c in nondiabetic populations has potential implications for the management and diagnosis of diabetes with increasing age.

It is also important to note the lack of significance in both regression models (assessing individual and cumulative MetS components) relating HbA1c to education and urban/rural location in all groups and the lack of association with household income except for the nondiabetic men group.

### From metabolic dysregulation to disease states and beyond

Using our approach that divided the population based on the presence of a disease state (diabetes), we were able to find significant associations between MetS components and HbA1c in nondiabetic populations and a lack of significance in most analyses of the diabetic population. This indicates significant associations and metabolic changes are not only occurring well below disease cutoff values but there are multiple metabolic influences that can be reflected using HbA1c prior to a disease state being established. This supports the potential for HbA1c to be used as a marker of metabolic health beyond diabetes diagnosis and management and may indicate a need for further investigation of other metabolic markers on an individual level before and after the development of a disease state.

Some MetS diagnostic criteria require a component of insulin resistance or indications of changes to glucose metabolism for diagnosis [38]. This was based on the previous understanding that MetS development always begins with insulin resistance before other MetS components accumulate [14]. Our results indicate that while insulin resistance may affect large portions of the population even prior to levels resulting in diabetes diagnosis, it is not necessarily required for other forms of metabolic dysregulation. In short, measurable insulin resistance is not necessary for MetS development. While not all those with multiple MetS components had indications of some forms of insulin resistance or changes to glucose metabolism it is still likely that some extent of insulin resistance is at play, but that the body is overcompensating via the other MetS components. The impact of MetS components is significant with or without changes to insulin production, sensitivity, and target tissue resistance or diabetes, further supporting that more than one pattern or process of metabolic dysregulation contributes to the development of MetS components and disease states over time.

While our findings indicate significant associations between MetS components and HbA1c, the proximate/physiological understanding of these associations, including the specific pathways that relate our variables, are not yet well understood. Furthermore, how metabolic risk factors contribute to not just an initial disease state, but also subsequent comorbidities, are also not well understood. This complexity is exemplified in the relationship between T2D and CVD. It is known that T2D is a major risk factor for CVD while in some individuals CVD may precede T2D development [48]. Additionally, treating these conditions independently may reduce risk or prevent developing the other disease [49]. While diseases may share common physiological mechanisms and shared clinical risk factors, the outcomes of these factors are widely varying between individuals and the pathways from shared conditions to disease state may develop either independently or via shared pathways [48, 50].

These findings provide further insight into the mismatch between evolved physiological mechanisms for metabolic regulation and contemporary environments. Extremely high rates of metabolic disorders are emerging specifically in areas with higher income, urbanism, high-calorie diets, overly hygienic environments, and sedentary lifestyles [51]. Many of these characteristics of post-agricultural/post-industrial environments directly increase the risk of MetS. For example, increased sedentary time generally leads to weight gain, and higher-calorie foods cause insulin bursts that wear the pancreas down [52]. Brain insulin resistance is an adaptive response to famine and harsh, cold environments, but in high-calorie, low-exercise circumstances, brain insulin resistance leads to systematic insulin resistance that leads to disease [53]. Beyond these direct pathways to disease, contemporary environments are also missing the richness in microbiota, helminths, and other symbionts that are required for optimal health. It is likely that the lack of microbiota in utero, at birth, in foods, and in the physical environment led to physiological systems that were designed to interact with a multitude of microbiota with only a fraction of microbiota to help balance and maintain complex systems in which they play an imperative role. This microbial disruption has effects on the immune system, which can bring about physiological changes that lead to MetS [54]. Importantly, the transition away from helminth infection has been identified as a potential contributor to metabolic diseases since they consume glucose and lipids, heighten insulin sensitivity, and increase immune activation [55]. While speculative, it is possible that some of the patterns associated with the diabetic group, such as increased inflammation, insulin resistance, and fewer associations between blood glucose and lipids, are the result of evolved responses to helminth infection that lead to disease states in the absence of parasite exposure.

The complex nature of NCD development necessitates future research to continue refining our knowledge of deleterious physiological changes that influence the patterns and timelines from metabolic dysregulation to initial NCD development and beyond. This includes assessing how lifestyle factors can impact physiological risk factors and disease development and progression. Future analysis should include additional confounding factors beyond those included in this study, such as diet and physical activity, to further examine the complex relationships between MetS components and HbA1c. Also, observing the development of MetS components over time and assessing the relationship to HbA1c would be a valuable future direction for research as well.

### Limitations

A main limitation of this study is the inability to assess the directionality of these associations due to the cross-sectional study design. Therefore, while we can ascertain that significant relationships exist between HbA1c and MetS components, a lack of directionality and longitudinal study design does not allow us to create causal pathways or understand these relationships beyond their existence. Additionally, the format of the THES does not provide a lot of detailed, qualitative, or ethnographic data due to the survey-based nature of the study. Additionally, diet and physical activity data were not included as these data have been shown to have low validity in WHO surveys.

A second limitation is that the gender variable was not self-reported; rather, the study interviewer estimated the participant’s “gender” based on their interpretation of appearance and then recorded “Male” or “Female”. For the present study, the variable was renamed from “sex” to “gender”, and the limitation of gender identification due to the methodology should be appreciated. It is also important to note that gender is not limited to men and women and thus fails to consider individuals who identify as non-binary, transgender, or other gender identities. Additionally, gender is not necessarily representative of biological sex, as it fails to consider intersex individuals. Our analysis separated women and men based upon the NCEP ATP III guidelines for MetS diagnosis, which has some differences based upon gender/sex; we used this as a proxy for hormonal differences that have marked impacts on the metabolic systems. However, it is important to note that while we separated males and females for our analyses, differences between males and females or men and women are not necessarily due to genetic factors but are also strongly influenced by numerous environmental factors (e.g., parasite exposure, diet, etc.) that influence biology [39].

A further limitation is that the present study was unable to differentiate between diabetes types. Increased HbA1c levels are present in both T1D and T2D, but the underlying pathological mechanisms that result in increased HbA1c are different between the two conditions. T1D and T2D have different pathophysiologies. T1D is a result of an autoimmune response that destroys pancreatic beta-cells, and T2D is due to sustained insulin resistance at a cellular level; the current study cannot distinguish between the causal mechanisms that might produce the observed outcomes in this study. Thus, it is possible that the inability to distinguish between the conditions may have increased the variance of our analyses and decreased the significance of our results. That said, it is expected that T2D is far more common in adult populations. Additionally, we are lacking other important types of data related to diabetes beyond type of diabetes, such as duration of disease and information related to diabetes management beyond medication, that may have provided a more nuanced understanding of variability in HbA1c in these groups.

In addition to biomarker values, medication use was also included in MetS criteria. Medication use variables were self-reported by participants, which has implications for the reliability of medication recall, use, and adherence.

Finally, HbA1c values can be affected by a variety of genetic, hematologic, and illness-related factors that influence erythropoiesis, hemoglobin structure, glycation, and erythrocyte destruction and have been found in previous studies to cause falsely high or low HbA1c results [27, 56] Some examples include recent blood loss, erythropoietin treatment, hemodialysis or transfusion, iron-deficiency anemia, kidney failure, and liver disease. Additionally, conditions that result in variation in hemoglobin type such as sickle cell anemia or a thalassemia may interfere with some HbA1c tests as well [27].

## Conclusions

Diabetes was documented in approximately 18% of individuals in this nationally representative sample in Tunisia, yet approximately 40% of those with diabetes had not received a clinical diagnosis, and therefore did not have access to medication. Of those with a diagnosis, 92% utilized medication, providing insights into healthcare and medication access. The diabetic men and women in this study had significantly higher (HbA1c, BP, TG, and WC) and lower (HDL) values, indicating worse metabolic health compared to the nondiabetic group. HbA1c was positively associated with more individual components of MetS in nondiabetic men (BP, HDL, and TG) and women (BP, TG, and WC) compared to diabetic men (TG) and women (none). HbA1c was positively associated with the number of cumulative MetS components in all groups, but especially in nondiabetic men and women.

Our findings provide insights into the growing prevalence of metabolic risk factors and diabetes in Tunisia, and potentially extend to other LMICs. By separating diabetic from nondiabetic participants, we are able to begin to assess how the degree of metabolic dysregulation is affected by the development of a disease state and have shown that it alters the relationship between MetS components and HbA1c. As the prevalence of metabolic and lifestyle risk factors and NCDs continue to increase we will require a better physiological understanding of how metabolic dysregulation leads to disease states over time.

## Data Availability

The datasets generated during the current study are available from the authors.

## Abbreviations

NCDs: noncommunicable diseases
MetS: Metabolic Syndrome
T1D: Type 1 Diabetes
T2D: Type 2 Diabetes
CVD: Cardiovascular Disease
TG: Triglycerides
HDL: High-density lipoprotein
WC: Waist circumference
BP: Blood pressure
HbA1c: Hemoglobin A1c
HICs: High-income countries
LMICs: Low- and middle-income countries

## References

[1] Non communicable diseases [cited 2022 Dec 1]. Available from: https://www.who.int/news-room/fact-sheets/detail/noncommunicable-diseases

[2] Grundy SM, Hansen B, Smith SC, Cleeman JI, Kahn RA. Clinical management of metabolic syndrome. Circulation. 2004;109(4):551–6. 14757684 10.1161/01.CIR.0000112379.88385.67

[3] Siu PM, Yuen QS. Supplementary use of HbA1c as hyperglycemic criterion to detect metabolic syndrome. Diabetol Metab Syndr. 2014;6:119. 25400701 10.1186/1758-5996-6-119 PMC4232661

[4] Cavero-Redondo I, Martínez-Vizcaíno V, Álvarez-Bueno C, Agudo-Conde C, Lugones-Sánchez C, García-Ortiz L. Metabolic syndrome including glycated hemoglobin A1c in adults: Is it time to change? J Clin Med. 2019;8(12):2090. 31805696 10.3390/jcm8122090 PMC6947260

[5] Kim H, Cho J, Jang J, Kang J, Seo M, Keon H, et al. Correlation between hemoglobin A1c and metabolic syndrome in adults without diabetes under 60 years of age. Korean J Fam Pract. 2017;7(1):60–5. 10.21215/kjfp.2017.7.1.60

[6] Dilley J, Ganesan A, Deepa R, Deepa M, Sharada G, Williams OD, et al. Association of A1C with cardiovascular disease and metabolic syndrome in Asian Indians with normal glucose tolerance. Diab Care. 2007;30(6):1527–32. 10.2337/dc06-2414 17351274

[7] Okosun IS, Annor F, Dawodu EA, Eriksen MP. Clustering of cardiometabolic risk factors and risk of elevated HbA1c in non-Hispanic White, non-Hispanic Black and Mexican-American adults with type 2 diabetes. Diabetes Metab Syndr. 2014;8(2):75–81. 24907170 10.1016/j.dsx.2014.04.026

[8] Khan HA, Sobki SH, Khan SA. Association between glycaemic control and serum lipids profile in type 2 diabetic patients: HbA1c predicts dyslipidaemia. Clin ExperMed. 2007;7(1):24–9. 10.1007/s10238-007-0121-3 17380302

[9] Saely CH, Aczel S, Marte T, Langer P, Hoefle G, Drexel H. The Metabolic Syndrome, Insulin Resistance, and Cardiovascular Risk in Diabetic and Nondiabetic Patients. J Clin Endocrinol Metab. 2005;90(10):5698–703. 16091486 10.1210/jc.2005-0799

[10] Singer DE, Nathan DM, Anderson KM, Wilson PWF, Evans JC. Association of HbA1c With Prevalent Cardiovascular Disease in the Original Cohort of the Framingham Heart Study. Diabetes. 1992;41(2):202–8. 1733810 10.2337/diab.41.2.202

[11] Esteghamati A, Zandieh A, Khalilzadeh O, Meysamie A, Ashraf H. Clustering of metabolic syndrome components in a Middle Eastern diabetic and non-diabetic population. Diabetol Metab Syndr. 2010;2(1):36. 20529329 10.1186/1758-5996-2-36 PMC2897775

[12] Mandal GK, Bhatt SA. Glycated hemoglobin levels in metabolic syndrome patients. Asian J Pharm Clin Res. 2020;13(11):101–3.

[13] Grundy SM, Cleeman JI, Daniels SR, Donato KA, Eckel RH, Franklin BA, et al. Diagnosis and management of the metabolic syndrome. Circulation. 2005;112(17):2735–52. 16157765 10.1161/CIRCULATIONAHA.105.169404

[14] Kahn R, Buse J, Ferrannini E, Stern M. The metabolic syndrome: Time for a critical appraisal: Joint statement from the American Diabetes Association and the European Association for the Study of Diabetes. Diab Care. 2005;28(9):2289–304. 10.2337/diacare.28.9.2289 16123508

[15] International Diabetes Federation. IDF Diabetes Atlas. 10th ed. Brussels, Belgium: International Diabetes Federation; 2021.

[16] Hall JE, Hall ME. Guyton and Hall Textbook of Medical Physiology. Elsevier Health Sciences; 2020. p. 1156.

[17] Liston A, Todd JA, Lagou V. Beta-cell fragility as a common underlying risk factor in type 1 and type 2 diabetes. Trends Mol Med. 2017;23(2):181–94. 28117227 10.1016/j.molmed.2016.12.005

[18] Felig P, Wahren J, Sherwin R, Palaiologos G. Amino acid and protein metabolism in diabetes mellitus. Arch Intern Med. 1977;137(4):507–13. 403871 10.1001/archinte.1977.03630160069014

[19] Saudek CD, Eder HA. Lipid metabolism in diabetes mellitus. Am J Med. 1979;66(5):843–52. 375725 10.1016/0002-9343(79)91126-4

[20] Action to Control Cardiovascular Risk in Diabetes Study Group, Gerstein HC, Miller ME, Byington RP, Goff DC, Bigger JT, et al. Effects of intensive glucose lowering in type 2 diabetes. N Engl J Med. 2008;358(24):2545–59. 18539917 10.1056/NEJMoa0802743 PMC4551392

[21] Diabetes Control and Complications Trial (DCCT). Results of feasibility study. The DCCT Research Group. Diab Care. 1987;10(1):1–19. 10.2337/diacare.10.1.1 2882967

[22] DeFronzo RA, Ferrannini E, Groop L, Henry RR, Herman WH, Holst JJ, et al. Type 2 diabetes mellitus. Nat Rev Dis Primers. 2015;1:15019. 27189025 10.1038/nrdp.2015.19

[23] Ducat L, Philipson LH, Anderson BJ. The mental health comorbidities of diabetes. JAMA. 2014;312(7):691–2. 25010529 10.1001/jama.2014.8040 PMC4439400

[24] Oguntibeju OO. Type 2 diabetes mellitus, oxidative stress and inflammation: examining the links. Int J Physiol Pathophysiol Pharmacol. 2019;11(3):45. 31333808 PMC6628012

[25] Dandona P, Aljada A, Chaudhuri A, Mohanty P, Garg R. Metabolic syndrome. Circulation. 2005;111(11):1448–54. 15781756 10.1161/01.CIR.0000158483.13093.9D

[26] Freeman VS. Glucose and hemoglobin A1c. Lab Med. 2014;45(1):e21-4. 10.1309/LMNSU432YJWCWZKX

[27] Gallagher EJ, Le Roith D, Bloomgarden Z. Review of hemoglobin A1c in the management of diabetes. J Diab. 2009;1(1):9–17. 10.1111/j.1753-0407.2009.00009.x 20923515

[28] Use of glycated haemoglobin (HbA1c) in the diagnosis of diabetes mellitus. Diab Res Clin Pract. 2011;93(3):299–309. 10.1016/j.diabres.2011.06.025 21820751

[29] Norris JM, Johnson RK, Stene LC. Type 1 diabetes—early life origins and changing epidemiology. Lancet Diab Endocrinol. 2020;8(3):226–38. 10.1016/S2213-8587(19)30412-7 PMC7332108 31999944

[30] Caballero AE, Ceriello A, Misra A, Aschner P, McDonnell ME, Hassanein M, et al. COVID-19 in people living with diabetes: An international consensus. J Diab Compl. 2020;34(9):107671. 10.1016/j.jdiacomp.2020.107671 PMC7336933 32651031

[31] Drucker DJ. Diabetes, obesity, metabolism, and SARS-CoV-2 infection: the end of the beginning. Cell Metab. 2021;33(3):479–98. 33529600 10.1016/j.cmet.2021.01.016 PMC7825982

[32] Rahmati M, Keshvari M, Mirnasuri S, Yon DK, Lee SW, Il Shin J, et al. The global impact of COVID-19 pandemic on the incidence of pediatric new-onset type 1 diabetes and ketoacidosis: A systematic review and meta-analysis. J Med Virol. 2022;94(11):5112–27. 35831242 10.1002/jmv.27996 PMC9350204

[33] Mattioli AV, Pinti M, Farinetti A, Nasi M. Obesity risk during collective quarantine for the COVID-19 epidemic. Obes Med. 2020;20:100263. 32838051 10.1016/j.obmed.2020.100263 PMC7282788

[34] Baekkeskov S, Hansen B, eds. Human diabetes: genetic, environmental and autoimmune etiology. Berlin, Heidelberg: Springer-Verlag; 1990.

[35] Ali O. Genetics of type 2 diabetes. World J Diabetes. 2013;4(4):114–23. 23961321 10.4239/wjd.v4.i4.114 PMC3746083

[36] Noncommunicable diseases country profiles 2018. [cited 2024 Apr 13]. Available from: https://www.who.int/publications-detail-redirect/9789241514620

[37] HEARTS D: diagnosis and management of type 2 diabetes. [cited 2023 Jul 13]. Available from: https://www.who.int/publications-detail-redirect/who-ucn-ncd-20.1

[38] Huang PL. A comprehensive definition for metabolic syndrome. Dis Model Mech. 2009;2(5–6):231–7. 19407331 10.1242/dmm.001180 PMC2675814

[39] DuBois LZ, Shattuck-Heidorn H. Challenging the binary: gender/sex and the bio-logics of normalcy. Am J Hum Biol. 2021;33(5):e23623. 34096131 10.1002/ajhb.23623

[40] Saidi O, Zoghlami N, Bennett KE, Mosquera PA, Malouche D, Capewell S, et al. Explaining income-related inequalities in cardiovascular risk factors in Tunisian adults during the last decade: comparison of sensitivity analysis of logistic regression and Wagstaff decomposition analysis. Int J Equity Health. 2019;18(1):177. 31730469 10.1186/s12939-019-1047-6 PMC6858762

[41] Rejaibi S, Cherif I, Ben Mansour N, Zoghlami N, Saidi O, Skhiri A, et al. Health care renunciation in Tunisian diabetic patients, 2016. Eur J Pub Health. 2020;30(Supplement 5):ckaa165.594. 10.1093/eurpub/ckaa165.594

[42] Kotti AB, Cherif A, Elloumi A. The social roots of health inequity in Tunisia: A preliminary study on the social determinants of health inequity. Adv Soc Sci Res J. 2021;8(4):576–93.

[43] Cherif I, Ben Mansour N, Rejaibi S, Zoghlami N, Saidi O, Skhiri A, et al. Prevalence, awareness and control of hypertension among Tunisian adults, 2016. Eur J Pub Health. 2020;30(Supplement 5):ckaa165.094. 10.1093/eurpub/ckaa165.094

[44] Pani LN, Korenda L, Meigs JB, Driver C, Chamany S, Fox CS, et al. Effect of Aging on A1C Levels in Individuals Without Diabetes: Evidence from the Framingham Offspring Study and the National Health and Nutrition Examination Survey 2001–2004. Diabetes Care. 2008;31(10):1991–6. 18628569 10.2337/dc08-0577 PMC2551641

[45] Davidson MB, Schriger DL. Effect of age and race/ethnicity on HbA1c levels in people without known diabetes mellitus: Implications for the diagnosis of diabetes. Diabetes Res Clin Pract. 2010;87(3):415–21. 20061043 10.1016/j.diabres.2009.12.013

[46] Roth J, Müller N, Lehmann T, Heinemann L, Wolf G, Müller UA. HbA1c and Age in Non-Diabetic Subjects: An Ignored Association? Exp Clin Endocrinol Diabetes. 2016;124(10):637–42. 27219885 10.1055/s-0042-105440

[47] Dubowitz N, Xue W, Long Q, Ownby JG, Olson DE, Barb D, et al. Aging is associated with increased HbA1c levels, independently of glucose levels and insulin resistance, and also with decreased HbA1c diagnostic specificity. Diabetic Medicine. 2014;31(8):927–35. 24698119 10.1111/dme.12459

[48] Fernandes Silva L, Vangipurapu J, Laakso M. The, “common soil hypothesis” revisited: risk factors for type 2 diabetes and cardiovascular disease. Metabolites. 2021;11(10):691. 34677406 10.3390/metabo11100691 PMC8540397

[49] Ceriello A, Motz E. Is oxidative stress the pathogenic mechanism underlying insulin resistance, diabetes, and cardiovascular disease? The common soil hypothesis revisited. Arterioscler Thromb Vasc Biol. 2004;24(5):816–23. 14976002 10.1161/01.ATV.0000122852.22604.78

[50] Stern MP. Diabetes and cardiovascular disease: The “common soil” hypothesis. Diabetes. 1995;44(4):369–74. 7698502 10.2337/diab.44.4.369

[51] Gluckman PD, Hanson MA, Low FM. Evolutionary and developmental mismatches are consequences of adaptive developmental plasticity in humans and have implications for later disease risk. Phil Trans Roy Soc B: Biol Sci. 2019;374(1770):20180109. 10.1098/rstb.2018.0109 PMC6460082 30966891

[52] Stearns SC. Evolutionary Medicine. Sunderland, Massachusetts: Sinauer Associates, Inc., Publishers; 2016.

[53] Tsatsoulis A, Mantzaris MD, Bellou S, Andrikoula M. Insulin resistance: An adaptive mechanism becomes maladaptive in the current environment—An evolutionary perspective. Metabolism. 2013;62(5):622–33. 23260798 10.1016/j.metabol.2012.11.004

[54] Rook G, Bäckhed F, Levin BR, McFall-Ngai MJ, McLean AR. Evolution, human-microbe interactions, and life history plasticity. Lancet. 2017;390(10093):521–30. 28792414 10.1016/S0140-6736(17)30566-4

[55] Gurven MD, Trumble BC, Stieglitz J, Blackwell AD, Michalik DE, Finch CE, et al. Cardiovascular disease and type 2 diabetes in evolutionary perspective: A critical role for helminths? Ev Med Pub Heal. 2016;2016(1):338–57. 10.1093/emph/eow028 PMC5101910 27666719

[56] Radin MS. Pitfalls in hemoglobin A1c measurement: When results may be misleading. J Gen Intern Med. 2014;29(2):388–94. 24002631 10.1007/s11606-013-2595-x PMC3912281

